# CpG preconditioning reduces accumulation of lysophosphatidylcholine in ischemic brain tissue after middle cerebral artery occlusion

**DOI:** 10.1007/s00216-020-02987-w

**Published:** 2020-10-19

**Authors:** Leonidas Mavroudakis, Susan L. Stevens, Kyle D. Duncan, Mary P. Stenzel-Poore, Julia Laskin, Ingela Lanekoff

**Affiliations:** 1 Department of Chemistry – BMC, Uppsala University, 75123 Uppsala, Sweden; 2 Department of Molecular Microbiology & Immunology, Oregon Health & Science University, Portland, OR 97239 USA; 3 Physical Sciences Division, Pacific Northwest National Laboratory, Richland, WA 99354 USA; 4 Department of Chemistry, Purdue University, West Lafayette, IN 47907 USA

**Keywords:** Ischemia, CpG preconditioning, Mass spectrometry imaging, Lysophosphatidylcholine, Phosphatidylcholine, Nano-DESI

## Abstract

Ischemic stroke is one of the major causes of death and permanent disability in the world. However, the molecular mechanisms surrounding tissue damage are complex and further studies are needed to gain insights necessary for development of treatment. Prophylactic treatment by administration of cytosine-guanine (CpG) oligodeoxynucleotides has been shown to provide neuroprotection against anticipated ischemic injury. CpG binds to Toll-like receptor 9 (TLR9) causing initialization of an inflammatory response that limits visible ischemic damages upon subsequent stroke. Here, we use nanospray desorption electrospray ionization (nano-DESI) mass spectrometry imaging (MSI) to characterize molecular effects of CpG preconditioning prior to middle cerebral artery occlusion (MCAO) and reperfusion. By doping the nano-DESI solvent with appropriate internal standards, we can study and compare distributions of phosphatidylcholine (PC) and lysophosphatidylcholine (LPC) in the ischemic hemisphere of the brain despite the large changes in alkali metal abundances. Our results show that CpG preconditioning not only reduces the infarct size but it also decreases the degradation of PC and accumulation of LPC species, which indicates reduced cell membrane breakdown and overall ischemic damage. Our findings show that molecular mechanisms of PC degradation are intact despite CpG preconditioning but that these are limited due to the initialized inflammatory response.

**Electronic supplementary material:**

The online version of this article (10.1007/s00216-020-02987-w) contains supplementary material, which is available to authorized users.

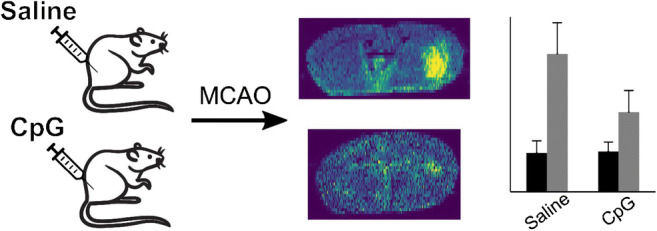

## Introduction

Stroke is the fifth leading cause of death in the USA, with a death occurring on average every 3 min 35 s [1]. Ischemic stroke, accounting for 87% of all strokes, occurs after the obstruction of blood vessels supplying blood to a region of the brain. During ischemic stroke, oxygen and nutrients, such as glucose, cannot reach the brain cells in this region, which leads to oxidative stress, ionic imbalance, inflammation, and ultimately apoptosis [2]. Furthermore, the depletion of adenosine triphosphate (ATP) in the ischemic area leads to disruption of the sodium-potassium pump. This ultimately results in accumulation of Na^+^ inside the cells and a concomitant decrease of K^+^ in the ischemic region [2–4]. While many events during ischemia are known, much remains to be learned, making it challenging but necessary to develop treatments to alleviate the negative complex biochemical mechanisms that are activated during stroke.

To elucidate the mechanisms and chain of events following ischemic injury, several stroke models have been developed [5]. These include both in vivo and in vitro models, where in the latter conditions are created to simulate the energy-deficient environment of ischemia. Since conditions are mimicked in human stroke by the occlusion of a major cerebral artery, middle cerebral artery occlusion (MCAO) is one of the most frequently used rodent animal models of ischemic stroke [5, 6]. Furthermore, this model has the advantage of producing an ischemic region in one hemisphere of the brain while leaving the other intact, which provides a damaged and control region within the same tissue section for analysis and evaluation.

Since stroke irreversibly damages brain tissue, prophylactic treatment has been proposed. Specifically, preconditioning initiates inflammatory responses that can limit tissue damage of subsequent ischemia [7]. Preconditioning can be achieved by inducing brief ischemic episodes [8] or by administrating a small dose of an otherwise harmful insult, such as an inflammatory mediator [9]. For example, a synthetic DNA oligonucleotide containing unmethylated cytosine-phosphate-guanine (CpG)-rich regions binds to a member of the Toll-like receptor (TLR) family, specifically TLR9, and initiates diverse immune effects [10]. The fact that TLRs are known to be involved in ischemia indicates their stimulation prior to ischemic stroke can provide neuroprotection [10]. In particular, systemic administration of CpG has been shown to provide neuroprotection against subsequent ischemic brain injury [10–12]. Although the exact mechanisms that follow pharmacological preconditioning are not fully understood, encouraging findings of mediated damage have been reported after prophylactic treatments [11].

Characterization of molecules involved in stroke and neuroprotective mechanisms can be achieved by mapping molecular signatures in regions of healthy and ischemic tissue. Mass spectrometry imaging (MSI) is a surface sampling technique where molecules from tissue surfaces can be analyzed without prior selection or staining and mapped to distinct cellular regions [13, 14]. By analyzing multiple distinct locations on the tissue, ion images of a selected ion can be generated to show molecular distributions. Several studies have used MSI, mainly matrix-assisted laser desorption/ionization (MALDI), to decipher the molecular alterations that take place in the ischemic brain region. For example, MALDI MSI demonstrated altered localization of phosphatidylcholines (PCs), lysophosphatidylcholines (LPCs), [15–20], and metabolites [21–23]. In addition, Mulder et al. recently utilized MALDI MSI to distinguish the ischemic core from the surrounding penumbra using lipid profiles [24]. Although MSI holds great promise for molecular elucidations directly from tissue, some obstacles need to be considered to enable determination of molecular changes in heterogeneous biological tissues. In particular, one effect of ischemia in tissue is the significantly altered abundance of alkali metals, which in combination with a decrease in pH poses a great challenge for interpreting results obtained with MSI. In particular, molecules in positive mode are detected as [M + H]^+^, [M + Na]^+^, and [M + K]^+^ ions and alteration in H^+^, Na^+^, and K^+^ concentrations in different parts of the analyzed tissue will affect the abundance of these detected species. By extension, increased abundances of [M + Na]^+^ ions and decreased signals of [M + K]^+^ ions of the same lipids have been observed in the ischemic hemisphere of the brain [15–17] compared to the healthy hemisphere, which makes it challenging to determine actual molecular alterations caused by ischemia.

Two strategies to remove artifacts originating from altered abundances of alkali metals in ischemic tissue and allow for representative molecular information in MSI have been presented. Firstly, Wang et al. developed a desalting method, where they rinsed the tissue section in ammonium acetate prior to MALDI MSI to remove sodium and potassium [25]. They were able to desalt the tissue without detectable delocalization of PCs or sphingomyelins (SMs), which enabled them to reveal lipidome changes induced by ischemia [20]. Secondly, Lanekoff et al. showed that accurate distributions of biomolecules in tissues are obtained by normalizing endogenous signals to a proper internal standard added to the nanospray desorption electrospray ionization (nano-DESI) solvent [26]. In comparison with the desalting approach, incorporation of internal standards does not require tissue pre-washing, or risk molecular delocalization.

Nano-DESI is a liquid extraction-based MSI technique in which molecules are desorbed into a continuously flowing solvent bridge formed between two fused silica capillaries [27]. The primary capillary delivers the solvent to the liquid bridge and the secondary capillary transfers the liquid and extracted molecules to the mass spectrometer inlet where molecules are ionized by nanoelectrospray ionization. Nano-DESI MSI [27–30] has been used to study nicotine distribution in rat brain tissue [31], metabolite diffusion from live bacterial colonies [32], imaging of lipids and metabolites in different types of tissues with moderate and high spatial resolution [33–35], MS/MS imaging of lipids and metabolites in mouse embryo implantation sites [36], and quantitative MSI of low abundance molecular species [37–39].

Herein we employ quantitative nano-DESI MSI to study distributions of PC and LPC species in mouse brain tissue sections from mice subjected to MCAO and reperfusion with and without CpG preconditioning. We show that CpG preconditioning not only reduces the visible size of the infarct but also suppresses the accumulation of LPC, which suggests that preconditioning decreases overall damage to the brain cells.

## Materials and methods

### Animal and tissue preparation

All animals were housed in a facility approved by the Association for Assessment and Accreditation of Laboratory Animal Care, met National Institute Health guidelines, and were approved by the Oregon Healthy and Science University Institutional Animal Care and Use Committee.

Mice were injected with either 200 μL of CpG ODN1826 (InvivoGen, San Diego, CA, USA) resulting in a dose of 0.8 mg/kg for preconditioning or saline vehicle by sub-cutaneous administration 72 h before MCAO.

Middle cerebral artery occlusion was induced in C57BL/6 mice (male, 8–12 weeks old, Jackson Laboratories, West Sacramento, CA, USA) as previously described [26]. Briefly, mice were anesthetized using 3% isoflurane and maintained with 1.5–2% throughout the operation. A silicone-coated 7–0 monofilament nylon surgical suture (Doccol, Redlands, CA, USA) was used to block the middle cerebral artery for 60 min. After removal of the filament, blood flow was restored for 2 h. Laser Doppler flowmetry (Transonic System Inc., Ithaca, NY, USA) was used to monitor the cerebral blood flow (CBF) and any mouse that failed to keep a CBF during occlusion of < 20% of baseline or did not reach > 80% of baseline after removal of the suture was not included in the study. A rectal thermometer-controlled heating pad and lamp (Harvard Apparatus, Holliston, MA, USA) was used to maintain the temperature at 37 ± 0.5 °C. Two hours following blood flow restoration, cold, heparinized saline was perfused into the mouse, the brain was removed, flash frozen in liquid nitrogen, and finally stored at − 80 °C. Coronal cryosections (CryoStar NX70, Thermo Fisher Scientific) of 12 μm were thaw-mounted onto regular glass slides.

### Nano-DESI MSI

The nano-DESI probe was assembled by placing two fused silica capillaries (50 × 150 μm, ID×OD, Polymicro Technologies, L.L.C. Phoenix, USA) in a capillary holder as described elsewhere [36]. To reduce the risk of scratching the sample surface, the primary capillary was beveled (Microbeveler 48000, World Precision Instruments, Inc.). Nano-DESI imaging was performed using a motorized X, Y, Z stage (Zaber Technologies Inc., Vancouver, BC) controlled by a custom-designed LabVIEW program, as described elsewhere [28]. For imaging experiments, the samples were moved in lines spaced by 200 μm under the nano-DESI probe at 40 μm s^−1^ while molecules were continuously desorbed into the solvent, at a scan rate of ~ 1.1 Hz, resulting in a pixel size of ~ 35 × 200 μm^2^ in the ion images. The solvent was delivered at 0.5 μL min^−1^ using a syringe pump (Legato 180, KD Scientific, MA, USA) and consisted of 9:1 methanol:water (v:v) and four internal standards. The four standards added to the nano-DESI solvent were: LPC 13:0, LPC 17:1, PC 12:0/13:0, and PC 21:0/22:6 at final concentrations of 3.55 μM, 3.23 μM, 4.69 μM, and 4.31 μM (Avanti Polar Lipids, Alabaster, AL, USA), respectively. These four standards have been used in a previous study and no overlaps with endogenous phospholipids were observed [26]. The nano-DESI source was coupled to an LTQ-Orbitrap XL mass spectrometer (Thermo Fisher Scientific) operated in positive mode at a mass resolution of 60,000 (*m/Δm*) at *m/z* 400. A voltage of 3 kV was applied to the primary capillary and the heated inlet was kept at 250 °C. Raw data were obtained with XCalibur (Thermo Fisher Scientific). Tissue sections from three mice preconditioned with saline and two mice preconditioned with CpG prior to MCAO were analyzed. Furthermore, three tissue sections from each mouse were imaged resulting in a total of nine and six replicates for saline and CpG-preconditioned mice, respectively. Lipid identification was based on accurate mass measurements and MS/MS (see Electronic Supplementary Material (ESM) Table S1 and Fig. S1).

### Data analysis

Raw data files were converted into mzXML files using MSConvertGUI [40] and then read into MATLAB R2019a (MathWorks, USA). In-house scripts were used to create ion images of molecules of interest by finding the closest peak within a mass accuracy tolerance of 5 ppm. Ion images were normalized to the appropriate internal standard [26] for quantitative MSI or to the total ion current (TIC) for comparing with internal standard normalized ion images. Data from regions of interest (ROI) of the ion images were extracted using scripts written in MATLAB. Specifically, two regions of interest were selected, the ischemic region and the healthy region. PC and LPC species were quantified in each pixel within each region, as described elsewhere [37]. The ROI of the ischemic half was selected based on the distribution of differentially abundant endogenous molecules and the optical image of the tissue section, and the ROI for the healthy half mirrored that of the ischemic one. All intensity values of zero were excluded from the data analysis. One-tail *t* tests were employed to determine statistically significant differences between the means of two groups at 95% confidence level. Error bars in figures represent one standard deviation of the mean from the combined replicates.

## Results and discussion

### Normalization enables visualization of LPC accumulation in ischemic region

**Fig. 1:**
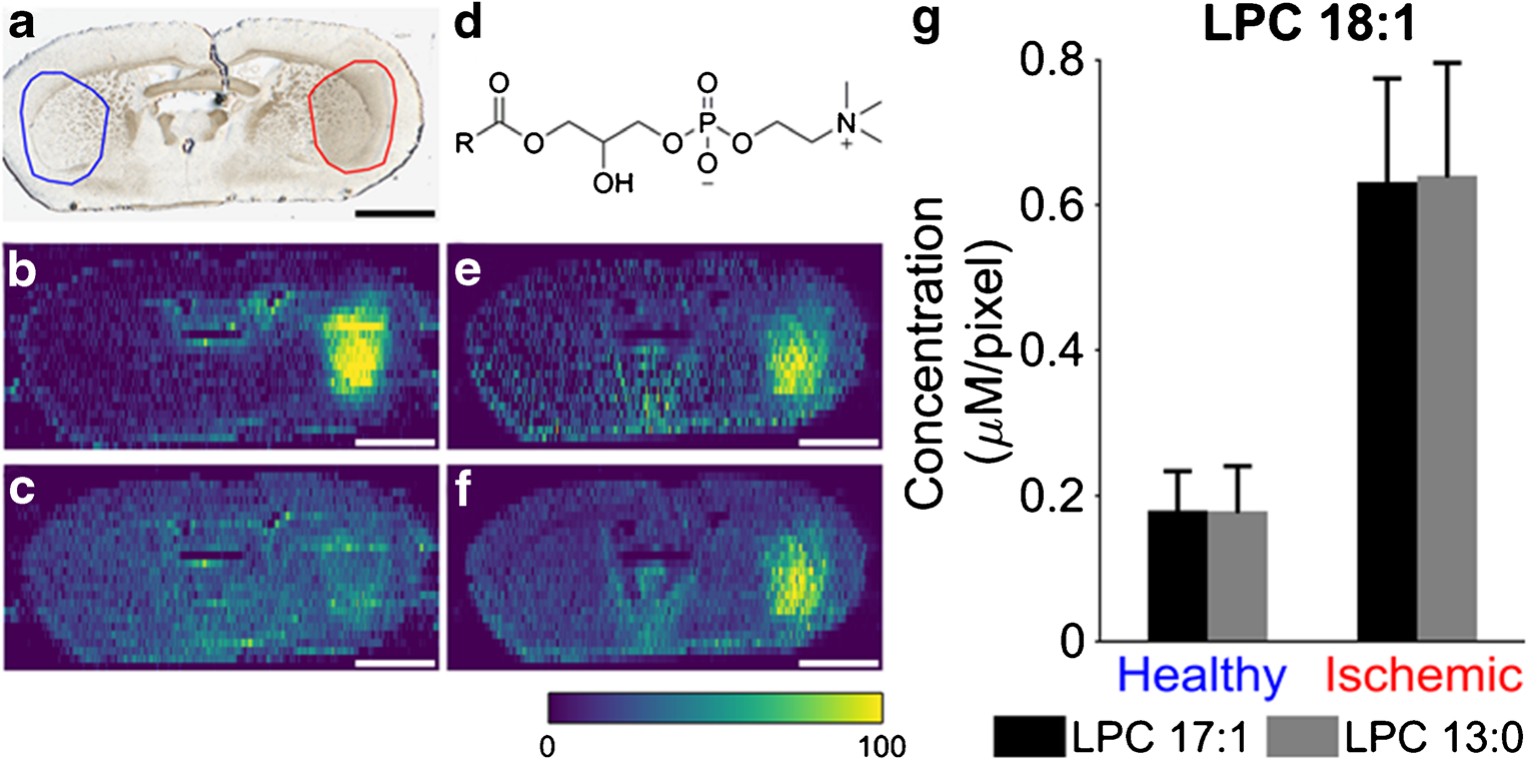
Normalization of endogenous LPC to TIC or internal standard. **a** Optical image of ischemic mouse brain tissue section. On the left with blue circle, the healthy half selection is indicated while the ischemic one is shown on the right with red circle. **b** TIC-normalized ion image of [LPC 18:1 + Na]^+^. **c** TIC-normalized ion image of [LPC 18:1 + K]^+^. **d** General structure of an LPC lipid. **e** Ion image of [LPC 18:1 Na]^+^ normalized to internal standard [LPC 17:1 + Na]^+^. **f** Ion image of [LPC 18:1 + K]^+^ normalized to internal standard [LPC 17:1 + K]+. **g** Average detected concentration of [LPC 18:1 + K]^+^ from healthy and ischemic ROIs normalized to [LPC 17:1 + K]^+^ (black bars) and [LPC 13:0 + K]^+^ (gray bars). Scale bars show 2 mm. Color bar scale ranges from 0 to 100% signal intensity. Error bars show standard deviation of pooled means from all replicates

Several reports have found that LPC is accumulating in the ischemic region of stoke [41]. However, the amount of accumulation is generally difficult to determine with MSI due to the significant increase in sodium and decrease in potassium concentration in this region, which results in different ion images depending on the selected adduct [3, 20]. A tissue section from a mouse subjected to MCAO is shown in Fig. 1a with the corresponding ion images of [LPC 18:1 + Na]^+^ and [LPC 18:1 + K]^+^, normalized to the total ion current (TIC), in Fig. 1b and c, respectively. TIC normalization is a typical strategy for presenting MSI data; however, it assumes that changes in the chemical composition in different cellular regions of the tissue do not affect ionization [42]. From Fig. 1b and c, it is clear that variations in sodium and potassium abundances in the ischemic region produce different TIC-normalized ion images of the sodium and potassium adducts of LPC 18:1. Specifically, there is a major increase in the signal of [LPC 18:1 + Na]^+^ in the ischemic region while the signal for [LPC 18:1 + K]^+^ is only slightly increased. Based on these TIC-normalized ion images, it is difficult to understand how ischemia affects the abundance of LPC 18:1. However, very similar ion images of [LPC 18:1 + Na]^+^ and [LPC 18:1 + K]^+^ are obtained when they are normalized to the respective adduct of the internal standard, LPC 17:1. Specifically, these ion images show increases of both [LPC 18:1 + Na]^+^ and [LPC 18:1 + K]^+^ in the ischemic region (Fig. 1e and f). From these ion images, it is thus clear that LPC 18:1 is increased in the ischemic region, regardless of selected adduct ion. This conclusion cannot be drawn from Fig. 1b and c where the ion images are normalized to TIC because Fig. 1b shows the additive effect of increased LPC 18:1 and increased Na in the ischemic region. Furthermore, Fig. 1c displays the canceling effect of increased LPC 18:1 with decreased K in the ischemic region. In addition, ion images of both adducts normalized to the internal standard provide a similar distribution of LPC 18:1 in other parts of the tissue section, which appears different from the distributions observed in TIC-normalized ion images. Thus, to compensate for matrix effects and enable molecular interpretation of the MSI data, normalization to the signal of a suitable internal standard is desired [26]. A proper internal standard has a similar ionization efficiency as the endogenous analyte. Therefore, isotopically labeled internal standards are often used in MSI [43], either applied onto tissue sections or introduced into the extraction solvent for liquid extraction techniques [44].

Although the internal standard LPC 17:1 is not isotopically labeled, its ionization efficiency is expected to be very similar to LPC 18:1. This is evidenced by the efficient compensation of the effect on species cationized on both sodium and potassium in Fig. 1e and f. Normalization to a different internal standard, LPC 13:0, also generates very similar ion images (ESM Fig. S2) suggesting that LPC 13:0 can be used as an internal standard. To evaluate whether ion images normalized to LPC 13:0 and LPC 17:1 provide identical quantitative information, the intensities of LPC 18:1, LPC 13:0, and LPC 17:1 were extracted from the healthy and ischemic regions of the mouse brain section for ROI analysis. The average detected concentration of LPC 18:1 in each region (*C*_end_) was calculated using Eq. 1 [37]:

1 $$ {C}_{\mathrm{end}}=\frac{I_{\mathrm{end}}}{I_{\mathrm{IS}}}\times {C}_{IS} $$

where *I*_end_ and *I*_IS_ are the mass spectrometric intensities of the endogenous lipid and internal standard, respectively, and *C*_IS_ is the concentration of the internal standard in the nano-DESI solvent. The average detected concentration for each ROI is presented in Fig. 1g using either LPC 13:0 or LPC 17:1 as the internal standard. Error bars in Fig. 1g include ROI data from all replicates. The almost identical calculated concentrations in healthy and ischemic regions indicate that LPC 13:0 and LPC 17:1 can be used interchangeably for both qualitative and quantitative analyses of LPC 18:1. Furthermore, either of the internal standards can be used for other endogenous LPC lipids, e.g., 16:0, 18:0, 20:4, and 22:6 (ESM Fig. S3) indicating that the potential differences in the ionization efficiency among LPC species are within the errors of the measurement. The effectiveness in using internal standards for MSI normalization of cationized species provides a new avenue for interrogating chemical alterations in stroke and neuroprotection by preconditioning.

### PC degradation is reduced in CpG-preconditioned mice

Preconditioning using CpG has been shown to mediate ischemic injury by activation of TLR9 [10, 45]. The damage associated with stroke is partially due to the inflammatory response and stimulation of TLRs, including TLR9 prior to ischemia, and provides neuroprotection in mice. Since neuroprotection with CpG is protein synthesis dependent, it takes at least 1 day to develop and lasts for up to 1 week [10]. Optical data indicate that 3 days after CpG administration, the infarcted area is reduced by 61% compared to administration with saline [10]. Thus, it is important to understand the differences between saline and CpG preconditioning on a molecular level.

TLRs are transmembrane proteins primarily located on the outside of the cellular plasma membrane surrounded by phospholipids. Phospholipids not only constitute a major component of cell membranes but are also an integral part of cellular functions such as ion transport, nutrient transportation, and signal transduction [46]. The major category of phospholipids localized at the outer leaflet of the plasma membrane is PC. Previous MSI studies focused on ischemic stroke have reported alteration in the PC abundances in the ischemic region of the tissue [15, 16, 20]. However, due to the significant alterations in cation abundances, the results are rather inconsistent. In particular, a decrease in the abundance of PC species in this region was reported based on the TIC-normalized distributions of [M + H]^+^ and [M + K]^+^ ions. Meanwhile, ion images of sodium adducts of PC species indicate that their abundance increases in the ischemic region. These discrepancies are eliminated when internal standards are used for normalization and quantification. Here, we employed this strategy to examine alterations in the abundance of PC and LPC species in MCAO-treated mice with and without CpG preconditioning. We examined biological replicates of the ischemic stroke brain tissue, in which MCAO occurred for 1 h and was followed by 2 h of reperfusion. For quantification of PC species, both PC 43:6 and PC 25:0 were added as internal standards to the extraction solvent, which enabled online pixel-by-pixel quantitation of the detected endogenous PC species regardless of the adduct formed. In the case of quantitation of PC lipids using PC 43:6 or PC 25:0 as internal standards, the internal standard normalized ion images did not show substantial differences. However, ROI analysis revealed statistically significant differences in the average detected concentration of PC 34:1 using the abovementioned PC internal standards (ESM Fig. S4). Differences in the ionization efficiency between these two PC internal standards have been observed in our previous study and carbon factors were used to account for the effect of the fatty acyl tail lengths on the ionization efficiency of PC species necessary for their accurate quantitation [47]. Since we are only reporting relative differences, both internal standards and either of the adducts may be used for quantification of all the detected endogenous PC species. However, for consistency, the remainder of the manuscript presents PC data normalized to the internal standard PC 43:6 using potassium adducts, to avoid potential isobaric interferences between sodiated and protonated adducts (ESM Table S2).

**Fig. 2:**
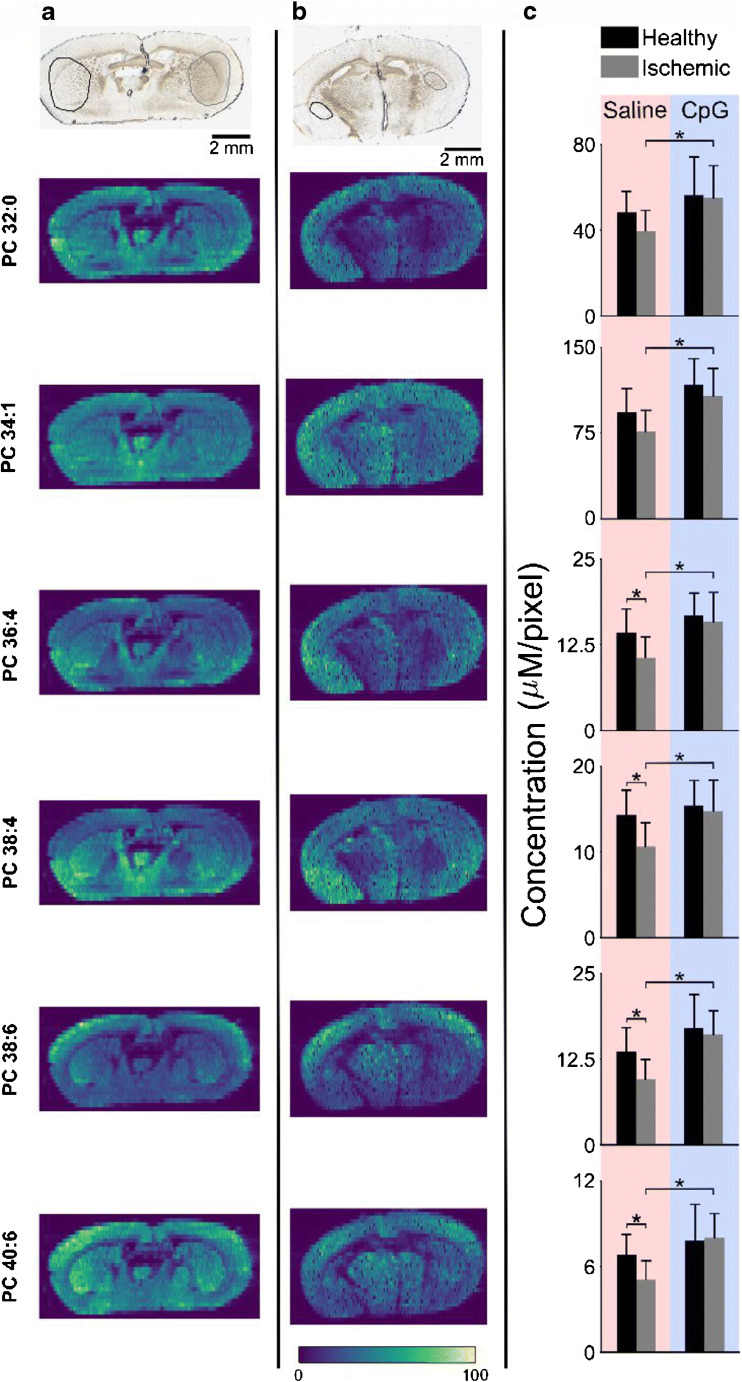
PC alterations in MCAO-treated preconditioned mice. Optical (top) and internal standard normalized ion images of six PC species in brain tissue section from MCAO-treated mouse preconditioned with **a** saline and **b** CpG. ROIs for the healthy and ischemic parts are marked in the optical images with black (left) and gray (right) circles, respectively. **c** ROI analysis showing average detected concentration of various PC species for saline- and CpG-treated mice. One-tail *t* tests were performed to determine statistically significant differences, *p < 0.02. All data shown depict potassium adducts of endogenous molecules normalized to the potassium adduct of internal standard PC 43:6. Color bar scale ranges from 0 to 100% signal intensity

The high abundance of PC in the plasma membrane of all cells generates ion images where PC is distributed over the entire tissue section. The distribution of six different endogenous PC species in tissue sections of MCAO-treated mice that were preconditioned with either saline or CpG are presented in Fig. 2a and b, respectively. Although the optical images clearly show a smaller infarcted area for the CpG-treated mouse, it is difficult to discern any substantial differences in PC abundances in this region. Instead, it appears that PC is more or less evenly spread over the entire tissue section, including the ischemic region (circled on the right side of the optical image). However, ROI analysis of the ischemic region and the corresponding healthy region, depicted in the optical images, reveals statistically significant decrease in the abundance of PC species in the ischemic region of saline-pretreated MCAO mice (Fig. 2c, saline). More specifically, one-tailed *t* tests show that PC 36:4, 38:4, 38:6, and 40:6 are significantly decreased in the ischemic region (*p* < 0.02), while the more abundant PC 32:0 and PC 34:1 display small but not statistically significant differences between the healthy and ischemic half of the mouse brain. The quantitative data in Fig. 2c, showing that PC 34:1 is detected with the highest relative concentration of all the six PC species followed by PC 32:0, are supported by previous report [48]. The detected concentration of the remaining four PC species is almost an order of magnitude lower. Therefore, we postulate that the relative decrease of the highly abundant PC 32:0 and 34:1 is not detectable.

We also observed higher detected concentrations of PC lipids in the ischemic region of CpG-treated compared to saline-treated mice (Fig. 2c, CpG). This could be attributed to less PC degradation in CpG preconditioning prior to stroke. For the PCs investigated here, no differences in abundance were observed between the healthy and ischemic half of brain tissue of CpG-preconditioned mice (Fig. 2c, CpG). Finally, the infarct size is much larger in the case of saline-treated mice compared to CpG preconditioned, as shown in the optical images in Fig. 2 (panels a and b) and also in previous reports [10, 11]. Collectively, our results indicate that PC degradation in the ischemic region is reduced due to CpG preconditioning.

PC lipids are targets for enzymatic degradation by phospholipase A_2_ (PLA_2_) and phospholipase C (PLC) and the detected decrease of PC in the ischemic region may be a result of the action of these enzymes. The currently accepted chain of events leading to PC degradation during ischemic stroke starts with decrease of ATP and release of glutamate from the synaptic pools of the neurons [46]. Next, glutamate activates the calcium-permeable NMDA receptors, which cause an influx of calcium into the cells. Subsequently, calcium activates calcium-dependent PLA_2_ that ultimately degrades PC lipids into LPC and free fatty acids [15, 26, 46]. The lower relative decrease of PC in CpG-treated mice suggests that the preactivation of TRL9 by CpG prior to stroke blocks the activation of this chain of events, and that PC degradation is indicative of cell membrane breakdown and subsequently irreversible ischemic damage.

### CpG preconditioning reduces LPC accumulation

**Fig. 3:**
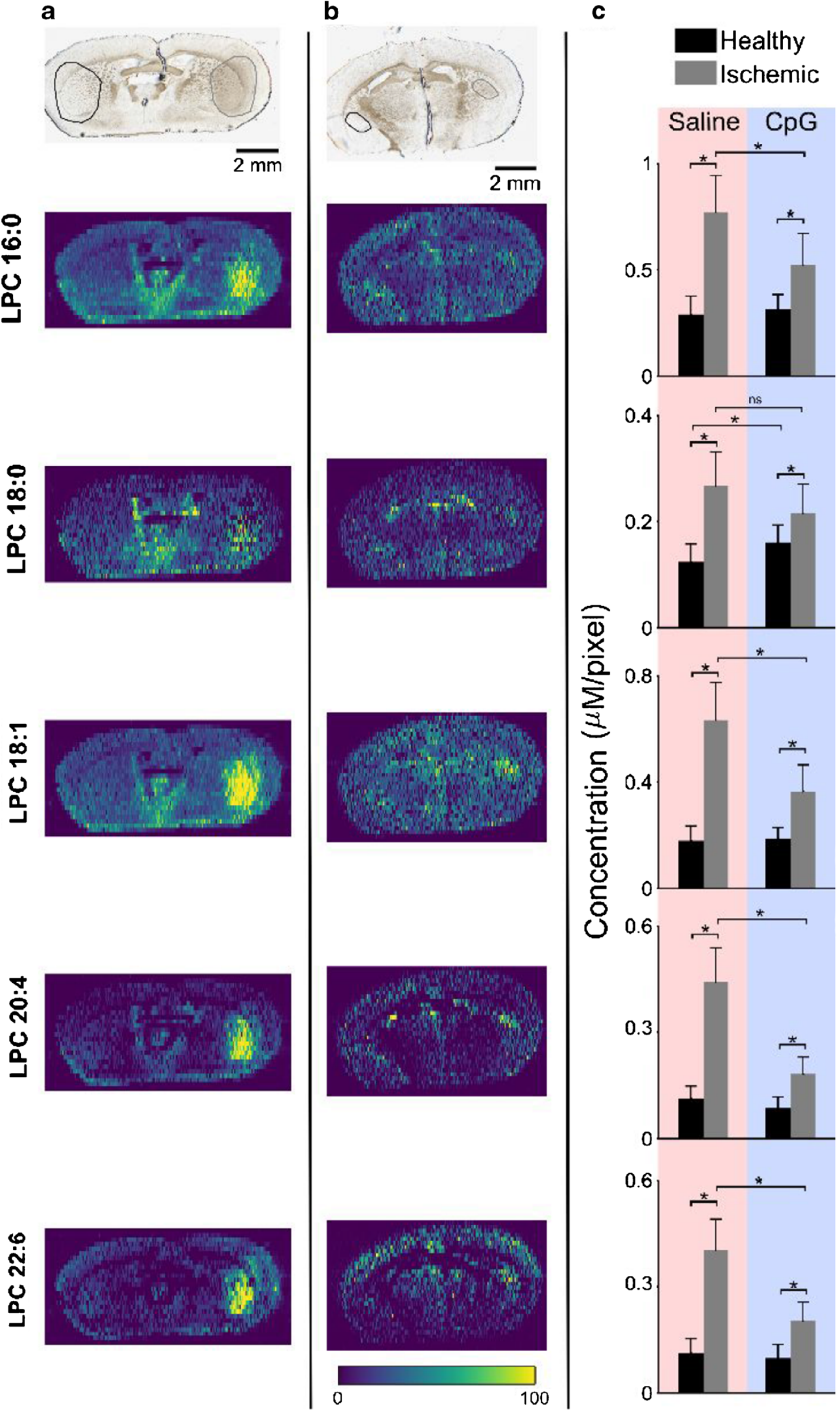
LPC alterations in MCAO-treated preconditioned mice. Optical (top) and internal standard normalized ion images of five LPC species in brain tissue section from MCAO-treated mouse preconditioned with **a** saline and **b** CpG. ROIs for the healthy and ischemic parts are marked in the optical images with black (left) and gray (right) circles, respectively. **c** ROI analysis showing average detected concentration of various LPC species for saline- and CpG-treated mice. One-tail *t* tests were performed to determine statistically significant differences, *p < 0.02; ns, not significant. All data shown depict potassium adducts of endogenous molecules normalized to the potassium adduct of internal standard LPC 17:1. Color bar scale ranges from 0 to 100% signal intensity

By including internal standards for LPC in the nano-DESI solvent, the impact of CpG preconditioning on the ischemic damage was examined based on the detected LPC species. According to the obtained ion images, the signals of LPC species in the ischemic region are noticeably higher for both saline- and CpG-preconditioned mice, as shown in Fig. 3a and b, respectively. However, the area of the brain showing an increased LPC signal is much smaller in the CpG-preconditioned mice. Particularly, the average number of pixels selected for the ROI is more than two times higher for the saline-treated mice (ESM Table S3). This suggests that the cellular damage, as indicated by the area of LPC accumulation, is much lower in CpG-preconditioned mice, which is in agreement with the decrease in the infarcted region observed using optical imaging [10]. Furthermore, the data shows that LPC 18:1 has the highest abundance in the center of the ischemic region, which shows that the degree of damage is more pronounced in the core region (ESM Fig. S5) [2, 24].

Quantitative ROI analysis on the accumulation of LPC further shows reduced damage of brain cells in CpG-preconditioned MCAO mice. Data displayed in Fig. 3c confirm that LPC is significantly increased in both saline- and CpG-treated mice. The detected LPC species included LPC 16:0, LPC 18:0, LPC 18:1, LPC 20:4, and LPC 22:6. This suggests that even though the damaged area is smaller, the chemical events that occur during ischemic stroke remain similar. However, the extent of cellular damage determined based on the enhanced abundance of LPC species is smaller in CpG-preconditioned mice. Specifically, although the fold change, calculated as the ratio of LPC concentrations in the ischemic over healthy region, ranges from 2.2 to 3.9 for saline-preconditioned mice, it ranges from 1.3 to 2.1 for CpG-preconditioned mice (ESM Table S4). Overall, the magnitude of cellular damage caused by ischemic stroke within the affected region is considerably smaller in CpG-preconditioned mice.

LPCs are the products of enzymatic breakdown of PCs by the action of phospholipases such as PLA_2_ and PLA_1_ [49]. While the general consensus is that PC lipids contain mostly C16 and C18 fatty acids in the sn-1 position and C20 and C22 polyunsaturated in the sn-2 position [50, 51], it has also been reported that PC has mainly saturated or monounsaturated fatty acids in the sn-2 position [46]. Our data show that LPC 16:0 and LPC 18:1 have the highest accumulation in the ischemic hemisphere compared to other LPCs. These phospholipids can be produced by the action of PLA_2_ on any PC having C16 or C18:1 fatty acids in the sn-1 position, and as depicted in Fig. 2a, PC 32:0 and PC 34:1 were the most abundant among all the reported PCs (ESM Table S5). However, we observed accumulated levels of polyunsaturated LPCs such as LPC 20:4 and LPC 22:6. It is possible that the action of PLA_1_ [20] leads to the formation of these phospholipids or they originated from the action of PLA_2_ on PCs having C20:4 and C22:6 in the sn-1 position.

Although the mechanisms responsible for preconditioning have not been established, it is speculated that they involve reprogramming of the brain response and the peripheral immune system to tolerate a subsequent stroke [9]. Our results showing the accumulation of LPC in both saline- and CpG-treated mice provide molecular level insights into CpG preconditioning. Specifically, our results suggest that the chain of chemical events during stroke, with accumulation of calcium in cells followed by activation of calcium-dependent phospholipases that generate LPC species, is not affected by CpG treatment. It is rather the extent of damage that is reduced in CpG-preconditioned mice.

## Conclusions

Due to the nature of MSI with ionization being dependent on the chemical composition in each spectrum, careful measures are needed to understand molecular alterations in complex biological systems. In particular, ischemic stroke causes a large shift in abundance of sodium and potassium. To avoid only monitoring effects based on adduct formation, we have shown that normalization to internal standards included in the nano-DESI solvent efficiently allows for imaging of endogenous species despite adduct formation, while also providing relative quantitation. Here, we have used our quantitative approach to study lipid alterations in brain tissue sections of mice subjected to MCAO with or without CpG preconditioning. Our results show that MCAO causes PC degradation to LPC in the ischemic region. In addition, while the damaged region of mice receiving CpG preconditioning is much smaller, the molecular mechanisms of PC degradation to LPC are intact. However, the degradation is significantly lower in preconditioned mice. Overall, our results provide evidence that prophylactic treatment with CpG reduces damage caused by ischemic stroke, both visually and molecularly, while molecular mechanisms of membrane breakdown are conserved.

## Electronic supplementary material

ESM 1 (PDF 1152 kb)
