# Supplementary material for: CpG preconditioning reduces accumulation of lysophosphatidylcholine in ischemic brain tissue after middle cerebral artery occlusion
Source: Anal Bioanal Chem. 2020 Oct 19;413(10):2735–45. doi: 10.1007/s00216-020-02987-w (PMC8007524; doi:10.1007/s00216-020-02987-w)
Supplement: Supplementary file 1 — (PDF 1152 kb) [file 216_2020_2987_MOESM1_ESM.pdf]

Analytical and Bioanalytical Chemistry

Electronic Supplementary Material

CpG preconditioning reduces accumulation of lysophosphatidylcholine in ischemic brain tissue after middle cerebral artery occlusion

Leonidas Mavroudakis, Susan L. Stevens, Kyle D. Duncan, Mary P. Stenzel-Poore, Julia Laskin, Ingela Lanekoff

Table of contents

Fig S1 MS/MS spectra of detected endogenous phospholipids.

Fig S2 Ion image comparison using different internal standard for normalization of endogenous LPC 18:1.

Fig S3 ROI concentration comparison using two different internal standards for normalization of endogenous LPC species.

Fig S4 Normalization of endogenous PC 34:1 using two different internal standards.

Fig S5 Ion image and line scan data showing dynamics of LPC accumulation.

Fig S6 PC alterations in MCAO treated mice.

Table S1 Annotated lipid species along with their observed and theoretical *m/z* and mass error in parts per million (ppm).

Table S2 Potential isobaric interferences on reported PC species as sodium adducts and mass resolving power required for peak separation.

Table S3 Number of pixels selected for each ROI.

Table S4 Average detected concentration (µM/pixel), standard deviation and fold change of LPC species in ischemic mouse brain preconditioned with saline or CpG.

Table S5 Average detected concentration (µM/pixel) and standard deviation of PC species in ischemic mouse brain preconditioned with saline or CpG

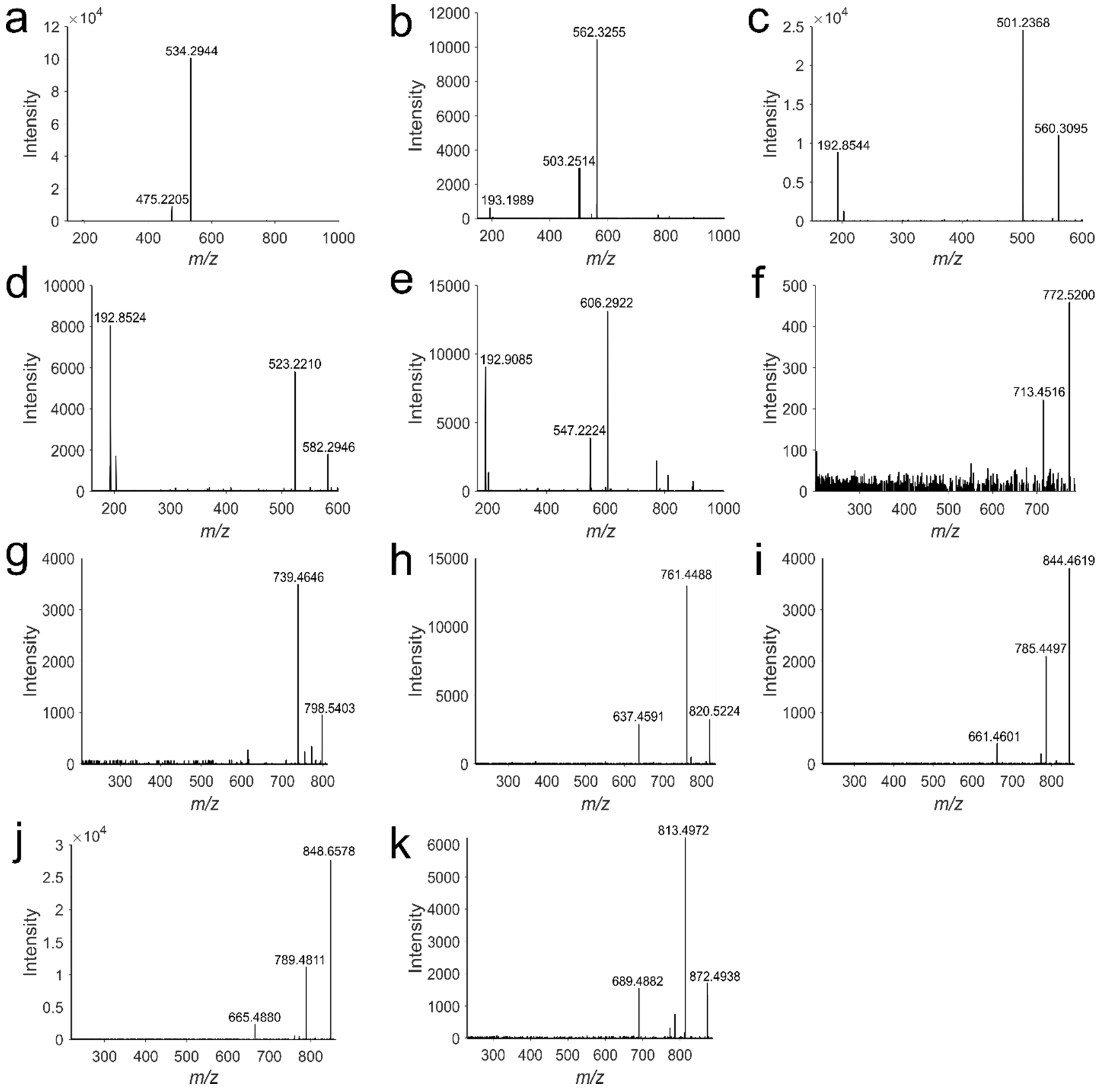

Fig. S1 MS/MS spectra of detected potassium adducts of endogenous phospholipids a) LPC 16:0 b) LPC 18:0 c) LPC 18:1 d) LPC 20:4 e) LPC 22:6 f) PC 32:0 g) PC 34:1 h) PC 36:4 i) PC 38:6 j) PC 38:4 k) PC 40:6. The loss of $N(CH_3)_3$ (59 mass units) is visible in all cases

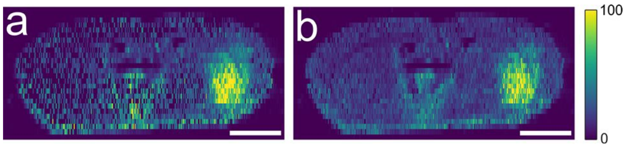

Fig. S2 Ion image comparison using different internal standard for normalization of endogenous LPC 18:1 a) Ion image of $[LPC\ 18{:}1 + Na]^{+}$ normalized to $[LPC\ 13{:}0 + Na]^{+}$. b) Ion image of $[LPC\ 18{:}1 + K]^{+}$ normalized to $[LPC\ 13{:}0 + K]^{+}$. Scale bar shows 2 mm. Colorbar scale ranges from 0 to 100 % signal intensity. For the optical image see Fig 1 of main text

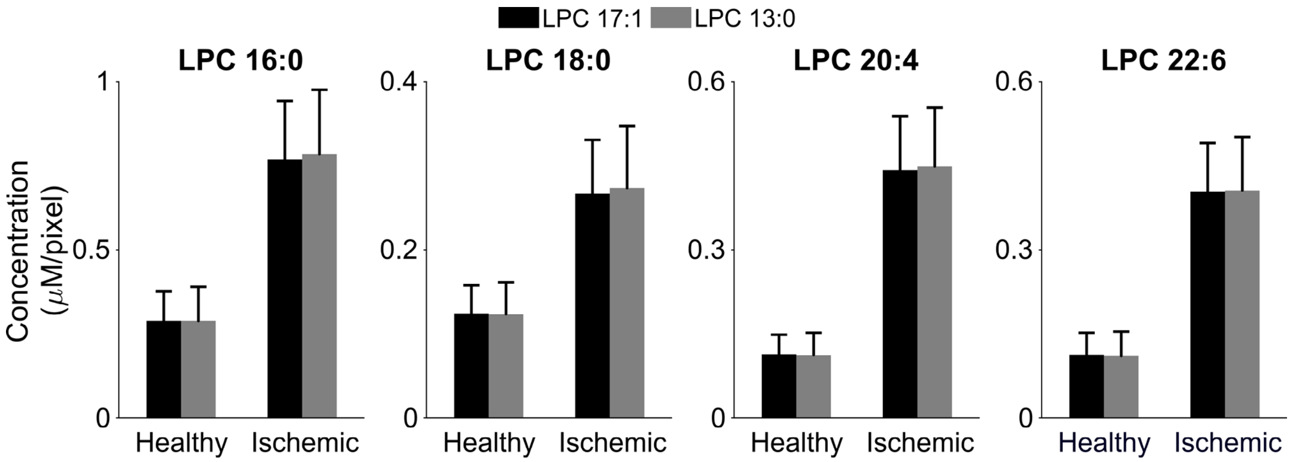

Fig. S3 ROI concentration comparison using two different internal standards for normalization of endogenous LPC species. Average concentrations of four endogenous LPC species in the healthy and ischemic part of mouse brain using LPC 17:1 (black bars) and LPC 13:0 (grey bars) as internal standard for quantitation. Potassium adducts were used for both endogenous and internal standard lipids

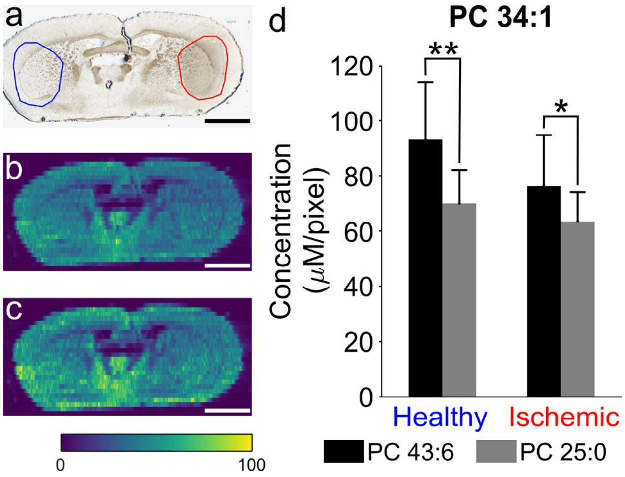

Fig. S4 Normalization of endogenous PC 34:1 using two different internal standards. a) Optical image of ischemic mouse brain tissue where the healthy and ischemic area are indicated by blue and red circle, respectively. b) Ion image of $[PC\ 34{:}1 + K]^+$ normalized to $[PC\ 43{:}6 + K]^+$. c) Ion image of $[PC\ 34{:}1 + K]^+$ normalized to $[PC\ 25{:}0 + K]^+$. d) Average concentrations of PC 34:1 extracted from ROI analysis in the healthy and ischemic part of the tissue section using PC 43:6 (black bars) and PC 25:0 (grey bars) as internal standards for quantitation. One tail t-test was performed to determine whether the means were statistically significant, $^*p < 0.05$ and $^{**}p < 0.01$. Color bar scale ranges from 0 to 100 % signal intensity. Scale bars show 2 mm

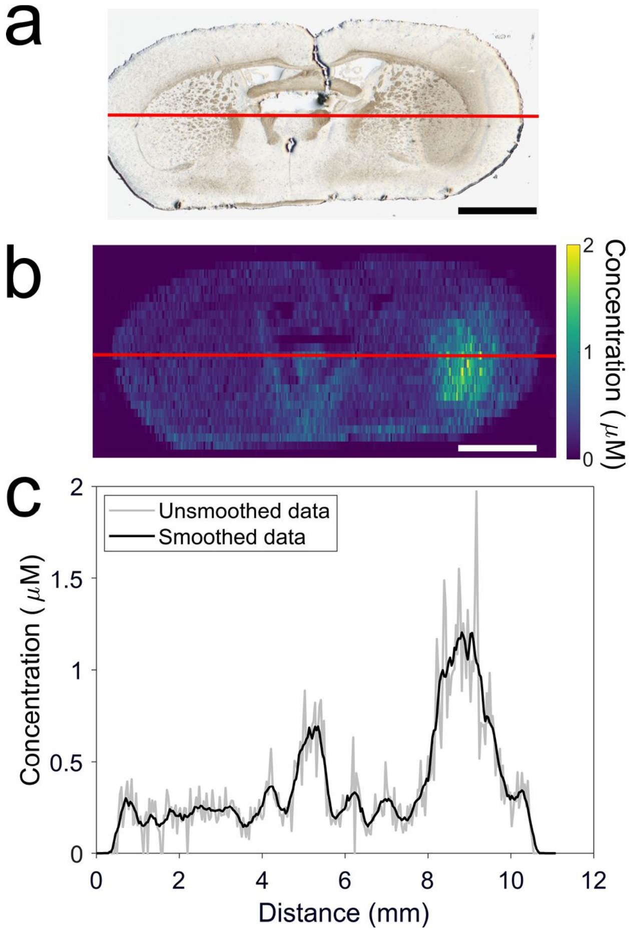

Fig. S5 Ion image and line scan data showing dynamics of LPC accumulation a) Optical image of ischemic mouse brain tissue section. b) Ion image of $[LPC\ 18:1 + K]^+$ normalized to standard $[LPC\ 17:1 + K]^+$. Normalized signal was converted to concentration (see main text). c) Line scan trace showing the detected concentration of LPC 18:1 when scanning from left to right of the tissue section. Red line in a and b show the line scan that is depicted in c. In c, both raw (grey line) and smoothed (black line) data are shown. Smoothing was done using rolling average of 9 data points. Scale bars show 2 mm

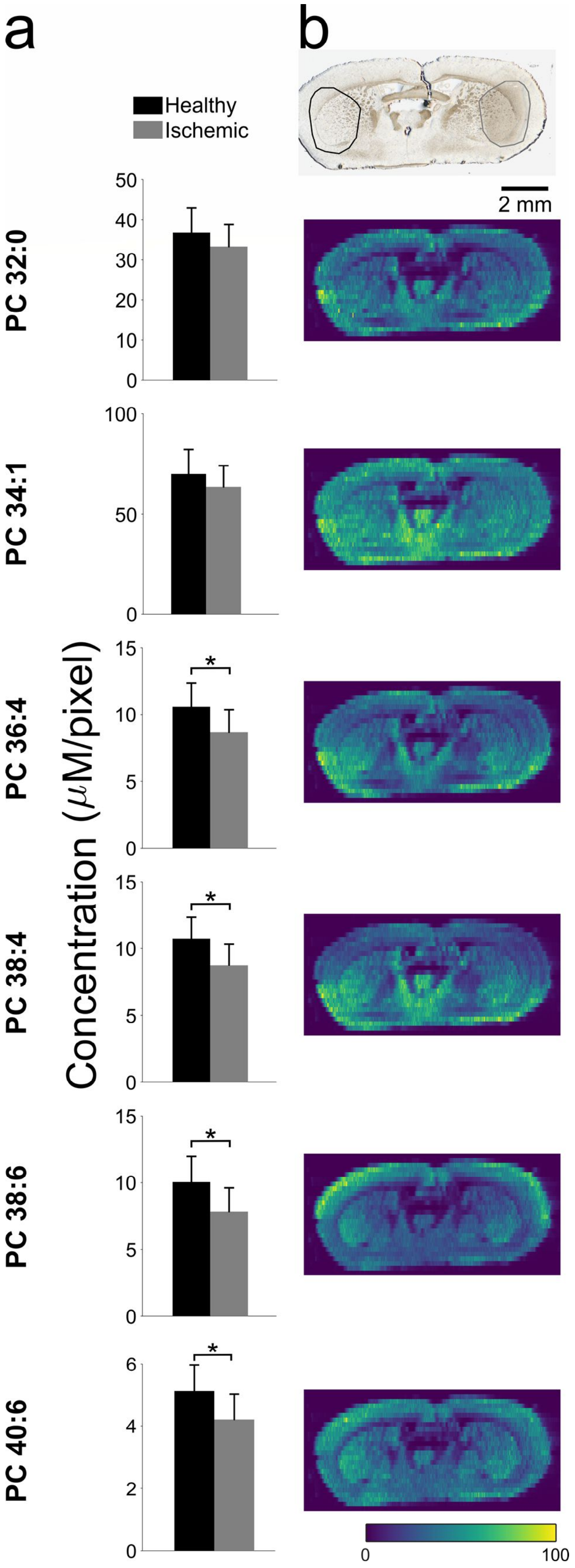

Fig. S6 PC alterations in MCAO treated mice. a) Average concentrations of selected PC species detected in healthy and ischemic part of mouse brain. One tail t-test was performed on the group means to determine statistically significant differences, *$p < 0.02$. Data were normalized to the internal standard PC 25:0 and potassiated adducts were used for both endogenous lipids and the internal standard. b) Optical images of ischemic mouse brain where the healthy and ischemic parts are indicated by black and grey circles, respectively. Internal standard normalized (PC 25:0) ion images of PC species detected in ischemic mouse brain. Color bar scale ranges from 0 to 100 % signal intensity

Table S1 Annotated lipid species along with their observed and theoretical *m/z* and mass error in parts per million (ppm)

Annotated species	Experimental *m/z*	Theoretical *m/z*	Mass error (ppm)
[LPC 13:0 + Na]$^{+}$ [a]	476.2746	476.2748	-0.42
[LPC 13:0 + K]$^{+}$ [a]	492.2486	492.2487	-0.20
[LPC 17:1 + Na]$^{+}$ [a]	530.3217	530.3217	0.00
[LPC 17:1 + K]$^{+}$ [a]	546.2956	546.2956	0.00
[LPC 16:0 + K]$^{+}$	534.2955	534.2956	-0.19
[LPC 18:0 + K]$^{+}$	562.3268	562.3269	-0.18
[LPC 18:1 + K]$^{+}$	560.3113	560.3113	0.00
[LPC 20:4 + K]$^{+}$	582.2957	582.2956	0.17
[LPC 22:6 + K]$^{+}$	606.2956	606.2956	0.00
[PC 25:0 + Na]$^{+}$ [a]	658.4420	658.4418	0.30
[PC 25:0 + K]$^{+}$ [a]	674.4159	674.4158	0.15
[PC 43:6 + Na]$^{+}$ [a]	898.6302	898.6296	0.67
[PC 43:6 + K]$^{+}$ [a]	914.6041	914.6036	0.55
[PC 32:0 + K]$^{+}$	772.5256	772.5253	0.39
[PC 34:1 + K]$^{+}$	798.5412	798.5410	0.25
[PC 36:4 + K]$^{+}$	820.5257	820.5253	0.49
[PC 38:4 + K]$^{+}$	848.5571	848.5566	0.59
[PC 38:6 + K]$^{+}$	844.5257	844.5253	0.47
[PC 40:6 + K]$^{+}$	872.5572	872.5566	0.69

[a]Standards included in the nano-DESI solvent

Table S2 Potential isobaric interferences on reported PC species as sodium adducts and mass resolving power required for peak separation

Lipid	Exact mass (amu)	Potential isobaric interference	Exact mass (amu)	Required resolving power
[PC 32:0 + Na]$^{+}$	756.5514	[PC 34:3 + H]$^{+}$ [a]	756.5538	315 000
[PC 34:1 + Na]$^{+}$	782.5670	[PC 36:4 + H]$^{+}$ [b]	782.5694	325 000
[PC 36:4 + Na]$^{+}$	804.5514	[PC 38:7 + H]$^{+}$ [c]	804.5538	335 000
[PC 38:4 + Na]$^{+}$	832.5827	[PC 40:7 + H]$^{+}$ [b]	832.5851	345 000
[PC 38:6 + Na]$^{+}$	828.5514	[PC 40:9 + H]$^{+}$ [d]	828.5538	345 000
[PC 40:6 + Na]$^{+}$	856.5827	[PC 42:9 + H]$^{+}$ [a]	856.5851	355 000

[a]Corresponding sodium and potassium adducts detected with low intensity
[b]Corresponding sodium and potassium adducts detected with high intensity
[c]Potassiated adduct detected with low intensity
[d]No sodiated and potasiated adducts detected

Table S3 Number of pixels selected for each ROI. Nine replicates (three mice and three sections from each mice) were used for saline preconditioning while six replicates (two mice and three sections from each mice) were used for CpG preconditioning

	Saline		CpG	
	Healthy half	Ischemic half	Healthy half	Ischemic half
**Number of pixels for ROI**	8858	8807	1900	1902
	9496	9464	1996	1973
	8879	8880	2928	2910
	6610	6609	2840	2839
	6558	6584	4081	4068
	5693	5720	3722	3718
	4291	4278		
	3416	3387		
	4555	4553		
**Average**	6484	6476	2911	2902
**SD**[a]	2209	2203	882	882

[a]Standard deviation

Table S4 Average detected concentration (μM/pixel), standard deviation and fold change of LPC species in ischemic mouse brain preconditioned with saline or CpG

		LPC 16:0	LPC 18:0	LPC 18:1	LPC 20:4	LPC 22:6
**Saline**	Healthy half	0.29 ± 0.09	0.12 ± 0.03	0.18 ± 0.05	0.11 ± 0.03	0.11 ± 0.04
	Ischemic half	0.77 ± 0.17	0.27 ± 0.06	0.63 ± 0.14	0.44 ± 0.10	0.40 ± 0.09
	Fold change[a]	2.66 ± 1.01	2.15 ± 0.78	3.51 ± 1.32	3.89 ± 1.45	3.58 ± 1.46
**CpG**	Healthy half	0.31 ± 0.07	0.16 ± 0.03	0.19 ± 0.04	0.09 ± 0.03	0.10 ± 0.04
	Ischemic half	0.52 ± 0.15	0.22 ± 0.05	0.36 ± 0.10	0.18 ± 0.05	0.20 ± 0.05
	Fold change[a]	1.68 ± 0.61	1.34 ± 0.44	1.95 ± 0.69	2.14 ± 0.97	2.06 ± 0.97

[a]Ratio of ischemic over healthy half, standard deviations are calculated using error propagation

Table S5 Average detected concentration (μM/pixel) and standard deviation of PC species in ischemic mouse brain preconditioned with saline or CpG

		PC 32:0	PC 34:1	PC 36:4	PC 38:4
**Saline**	Healthy half	48.29 ± 9.67	93.11 ± 20.80	14.30 ± 3.39	14.27 ± 2.91
	Ischemic half	39.67 ± 9.51	76.24 ± 18.49	10.60 ± 3.00	10.61 ± 2.80
**CpG**	Healthy half	56.31 ± 17.81	116.80 ± 22.99	16.70 ± 3.26	15.41 ± 2.89
	Ischemic half	54.98 ± 15.04	107.30 ± 24.06	15.82 ± 4.27	14.74 ± 3.58

Table S5 (continued)

		PC 38:6	PC 40:6
**Saline**	Healthy half	13.60 ± 3.46	6.82 ± 1.42
	Ischemic half	9.56 ± 2.91	5.07 ± 1.34
**CpG**	Healthy half	16.99 ± 4.88	7.78 ± 2.55
	Ischemic half	16.07 ± 3.48	7.99 ± 1.69
